# Bonding the foe – NETting neutrophils immobilize the pro-inflammatory monosodium urate crystals

**DOI:** 10.3389/fimmu.2012.00376

**Published:** 2012-12-10

**Authors:** Christine Schorn, Christina Janko, Veit Krenn, Yi Zhao, Luis E. Munoz, Georg Schett, Martin Herrmann

**Affiliations:** ^1^Institute for Clinical Immunology and Rheumatology, Department of Internal Medicine III, University of Erlangen-NurembergErlangen, Germany; ^2^Institute of PathologyTrier, Germany; ^3^Department of Rheumatology and Immunology, West China Hospital, Sichuan UniversityChengdu, China

**Keywords:** NETosis, NETs, MSU, opsonins, inflammation, ROS, gout

## Abstract

In the presence of sodium, uric acid from purine metabolism precipitates as monosodium urate (MSU) needles and forms renal calculi or causes gouty arthritis in kidneys and joints, respectively. The latter is characterized by red, hot, and swollen arthritic joints. Here we report the *in vitro* effect of MSU crystals on blood granulocytes and analyze their contribution to granuloma formation and neutrophil extracellular traps (NETs) formation (NETosis) in synovial fluid of patients with gouty arthritis *in vivo*. We observed that MSU crystals induce NETosis *in vitro* in a reactive oxygen species (ROS)-dependent manner. Indeed, blocking ROS (e.g., the oxidative burst) by various anti-oxidants partially inhibited NETosis induced by MSU crystals. Analyses of synovial fluids and of tissue sections of patients suffering from gout revealed that NETs are also formed *in vivo*, especially during acute gouty flares and/or granuloma formation. Since prolonged exposure to NETs carries the risk for the development of chronic inflammation we also studied the opsonization of NETs, as a prerequisite for their clearance. The established dead cells’ opsonins C3b, galectin-9, and CRP decorated the residual dead cells’ corpses and opsonized these for disposal. Surprisingly, all three soluble pattern recognizing molecules spared the spread NET structures. We conclude that (i) MSU crystals are strong inducers of ROS-dependent NETosis and (ii) that the prolonged presence of NET-pathogen or NET-crystal aggregates observed in patients with systemic autoimmunity, especially in those with low serum DNase-1 activity, cannot be compensated by CRP, complement, and galectin-mediated phagocytic clearance.

## INTRODUCTION

Uric acid is the final product of the purine catabolism in humans. Most uric acid dissolves in blood and is excreted in urine via the kidneys. Reduced removal of uric acid or increased purine breakdown (caused by purine rich food or massive tissue damage and cell death) leads to elevated levels of circulating uric acid. If the latter exceeds its solubility limit (approximately 6.8 mg/dl), it precipitates in sodium containing fluids as monosodium urate (MSU) crystals in the extracellular milieu (Schorn et al., 2009). The accumulation of MSU crystals in joints is the etiological reason for the painful inflammatory attacks in patients suffering from gout.

Once formed, MSU crystals act as endogenous danger signal stimulating the innate immune system to elicit an inflammatory response. Depositions of MSU crystals are often accompanied by massive leukocyte infiltrations (Popa-Nita and Naccache, 2010). After phagocytosis by monocytes, MSU crystals activate the NALP3 inflammasome with concomitant IL-1β release by monocytes (Schorn et al., 2010, 2011). Along with chemotactic factors, IL-1β attracts and activates neutrophils and other immune cells, which leave the blood vessels and migrate along the chemotactic gradient toward the MSU depots. In response to chemical stimuli, microbial pathogens or MSU crystals, neutrophils, eosinophils, basophils, and mast cells have been shown to release DNA into the extracellular milieu (Brinkmann et al., 2004; von Kockritz-Blickwede et al., 2008; Schorn et al., 2012). If these structures are released from neutrophils they are usually referred to as neutrophil extracellular traps (NETs). In analogy, for extranuclear DNA released from eosinophils, basophils, and mast cells the terms EETs, BETs (Schorn et al., 2012), and MCETs (von Kockritz-Blickwede et al., 2008) may be used, respectively.

Neutrophil extracellular traps are composed of DNA, histones, granular enzymes, and anti-microbial proteins, ejected in response to strong phagocytic stimuli (Brinkmann et al., 2004; Urban et al., 2006). Thus, NETs provide a powerful arsenal of anti-microbial factors in high local concentrations. Beside MSU crystals PMA, bacteria or fungi are potent NETs inducers (Brinkmann and Zychlinsky, 2012). The sticky DNA fibers of the NETs bind and immobilize pathogens and thus inhibit their further spreading. Therefore, beside phagocytosis, intraphagosomal killing and secretion of anti-microbials, NET formation is a crucial mechanism to control and fight various classes of pathogens. It has been shown that neutrophils exert protective effects in endotoxemia and sepsis by releasing NETs and thus capturing circulating bacteria from the blood stream (McDonald et al., 2012).

When cells release NETs they undergo a cell death pathway called “NETosis,” morphologically distinct from all other cell death pathways like apoptosis and necroptosis and others (Fuchs et al., 2007). However, some features of other cell death pathways may also operate during NETosis. The following steps are required for NETosis: (i) the generation of reactive oxygen species (ROS) by the nicotinamide adenine dinucleotide phosphate (NADPH) oxidase (Fuchs et al., 2007), (ii) the translocation of neutrophil elastase and myeloperoxidase from the granules to the nuclei, (iii) the conversion of arginine residues to citrulline in core histones by protein arginine deiminase 4 (PAD4), being necessary for proper decondensation of chromatin before release as NETs (Neeli et al., 2008), and (iv) the rupture of the plasma membrane (Brinkmann and Zychlinsky, 2012).

In this study, we analyzed the role of ROS in MSU-induced NET formation. We observed in accordance with published data for bacterial-induced NETosis, that anti-oxidants also inhibit the formation of NETs after stimulation of neutrophils with MSU. Extracellular chromatin containing structures with the appearance of NETs can also be detected in tissue sections and synovial fluids of patients suffering from gout. Since NETs are not bound by opsonins we consider NETs as problematic prey for the opsonin-mediated clearance and a potential antigenic structure for etiology and pathogenesis of systemic autoimmunity.

## MATERIALS AND METHODS

### HUMAN MATERIAL

All analyses of human material were performed in full agreement with institutional guidelines. We prepared paraffin sections of gouty granulomas from patients suffering from gouty arthritis and compared them with sections of synovial controls (*n* = 5). We analyzed synovial fluids (anti-coagulated with 20 U/ml heparin) from patients suffering from gout and patients with MSU-free synovitis. Heparinized (20 U/ml) venous blood was obtained from normal human blood donors. Autologous plasma was extracted from heparinized venous blood by centrifugation at 3400 *g* for 10 min (Rotina 46, Hettich). CRP was isolated from human sera of patients with bacterial infections by affinity chromatography using phosphocholine sepharose (Thermo Fisher). We labeled CRP with FITC according to the instructions of the manufacturer (Sigma-Aldrich).

### ISOLATION OF PMN FROM HUMAN BLOOD

Polymorphonuclear neutrophils (PMN) were isolated from heparinized blood (20 U/ml) by ficoll density gradient centrifugation using standard protocols. Shortly, PMN were collected from the buffy coats. Residual erythrocytes were eliminated by hypotonic lysis. Viable cells were counted by trypan blue exclusion in a Neubauer chamber. The cell count was adjusted to 2 to 5 × 10^6^ PMN/ml. PMN were cultured in autologous active plasma containing functional complement.

### OPSONIN BINDING TO NETs

Isolated PMN were incubated with 200 μg/ml MSU crystals for 5 h at 37°C and then fixed with 1% paraformaldehyde. Cytospins were prepared and treated for 30 min at 37°C with fresh human plasma to allow complement binding. NETs were visualized by propidium iodide (PI) staining employing fluorescence microscopy. The binding of opsonins was analyzed by CRP-FITC, anti-C3b-FITC (Dako), and biotinylated Gal-9 plus streptavidin-FITC (Sigma-Aldrich). As control we used an anti-dsDNA antibody plus anti-human IgG-FITC (Southern Biotech).

### HISTOLOGY

DNA was stained for 30 min with 4^′^,6-diamidino-2-phenylindole (DAPI; Invitrogen GmbH) or with PI (Sigma-Aldrich) at 1 or 4 μg/ml, respectively. After washing, the samples were analyzed by fluorescence microscopy using standard filter sets.

### INTRACELLULAR ROS PRODUCTION

Dichlorofluorescein-diacetate (DCFH-DA) is freely permeable across cell membranes. Inside the cells, the acetate moiety is cleaved off by esterases to yield the membrane-impermeable non-fluorescent DCFH. In the presence of ROS, DCFH is oxidized and forms the fluorescent DCF. Anti-coagulated blood was incubated with 10 μM DCFH-DA (Sigma-Aldrich) at 37°C. After 30 min, 1 mg/ml MSU crystals were added, and the samples were incubated at 37°C for up to 8 h. After erythrocyte lysis, the intracellular DCF fluorescence of the leukocytes was recorded by flow cytometry.

### NET FORMATION IN PRESENCE OF ANTI-OXIDANTS

Whole blood was incubated with 1 mg/ml MSU or co-incubated with 250 μM butylated hydroxytoluene (BHT), 200 μM butylated hydroxyanisole (BHA), or 300 μM ascorbic acid (all from Sigma-Aldrich) for 5 h at 37°C. After the lysis of erythrocytes and solubilization of MSU crystals, cytospins were prepared and stained with DAPI.

### CYTOSPINS

We centrifuged 2 × 10^5^ cells at 850 *g* for 10 min (Rotina 46, Hettich) with a cytospin cuvette on glass slides (Thermo Fisher). After draining the supernatants, the cells were centrifuged for 2 min at 2000 *g*. The fixed cells were then analyzed by light and fluorescent microscopy.

### PREPARATION OF MSU CRYSTALS

For the synthesis of MSU crystals, a solution of 10 mM uric acid and 154 mM NaCl (both from Merck KGaA) was adjusted to pH 7.2 and agitated for 3 days. The resulting crystals were pelleted, washed with ethanol, and dried under sterile conditions. The crystals were deprived of lipopolysaccharides by heating to 180°C for 2 h and can be stored in PBS (pH 7.0) at ambient temperature for at least 1 month.

### LYSIS OF ERYTHROCYTES, SOLUBILIZATION OF CRYSTALS, AND FLOW CYTOMETRY

In whole blood assays, the erythrocytes were automatically lysed using a TQprep Workstation (Beckman Coulter) before measurement with a Gallios cytofluorometer (Beckman Coulter). The data were analyzed with the Kaluza software 1.2 (Beckman Coulter). Electronic compensation was used to eliminate bleed-through fluorescence. The erythrocyte lysis conditions also solubilized non-ingested MSU crystals.

### STATISTICAL ANALYSIS

We performed statistical analyses with SPSS PASW statistics 18. The results are represented as mean ± SD of at least three and up to five independent experiments. Student’s *t*-test or an analysis of variance for repeated measurements was used. The data were considered significant and highly significant for *P*-values <0.05 and <0.01, respectively.

## RESULTS

### MSU CRYSTAL-INDUCED NETOSIS DEPENDS ON ROS

**FIGURE 1:**
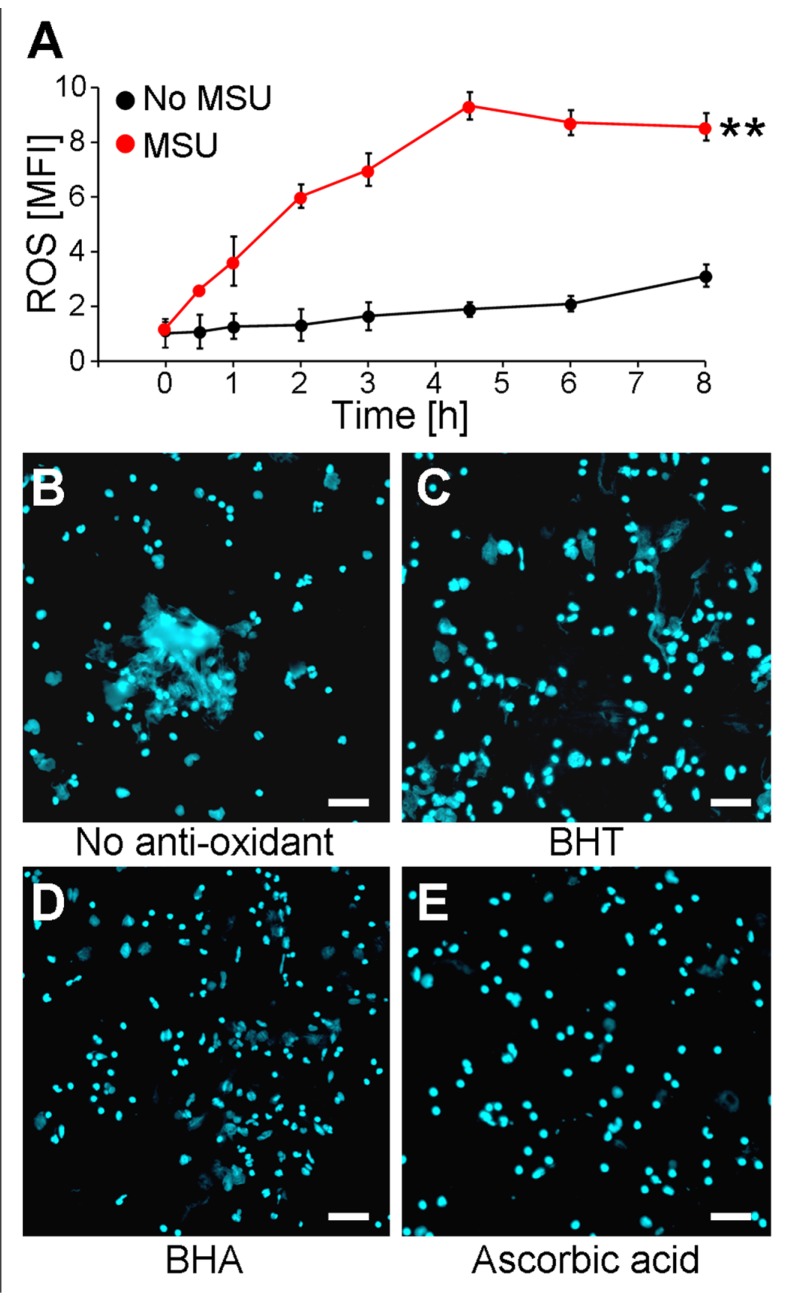
**MSU-induced NET formation depends on oxidative stress (ROS)**. **(A)** Whole blood was treated with or without MSU (*n* = 3 per group) and analyzed for ROS production by cytofluorometry. MSU crystals induce oxidative stress (ROS) in neutrophils. **P < 0.01. **(B–E)** Whole blood was incubated with MSU in the absence **(B)** or the presence of BHT **(C),** BHA **(D)**, or ascorbic acid **(E)**. Cytospins were stained for extranuclear DNA. NET formation by neutrophils after incubation with MSU was inhibited by the anti-oxidants BHT **(C)**, BHA **(D)**, and ascorbic acid **(E)**. All experiments were performed at least three times. Scale bars, 100 μm.

It was previously shown that NET formation induced by chemicals, various pathogens, or cytokines is strongly augmented by the presence of ROS generated by activated NADPH oxidase (von Kockritz-Blickwede et al., 2008). Since we observed that MSU crystals are potent inducers of NETs, we analyzed if MSU-initiated NETosis is accompanied by and dependent on the formation of ROS in human whole blood *ex vivo* cultures. The ROS productions of individual cell populations were determined by DCF fluorescence. DCFH-DA is commonly used to quantify ROS on a single cell level in flow cytometry. DCFH-DA passively penetrates individual cells and is trapped as DCFH in the cytoplasm after deacylation by intracellular esterases. In the presence of ROS the latter forms the highly fluorescent DCF, which can be detected in cytofluorometry. Human anti-coagulated whole blood was incubated with 10 μM DCFH-DA at 37°C in the presence and absence of MSU crystals. Already 30 min after the addition of the crystals ROS was to be detected. The DCF fluorescence reached its maximum after 4.5 h. In the absence of MSU the DCF fluorescence was virtually stable for up to 8 h (**Figure 1A**).

The incubation of human whole blood *ex vivo* cultures with 1 mg/ml MSU for 5 h at 37°C resulted in the formation of extended supercellular structures containing extranuclear DNA as detected by DAPI staining (**Figure 1B**). The extracellular DNA was confirmed as NETs employing stainings against neutrophil elastase, myeloperoxidase, and histones (not shown). To analyze if the formation of NETs requires ROS, the experiment was also performed in the presence of the potent anti-oxidants BHA, BHT, and ascorbic acid. The treatment with the various anti-oxidants did not influence the cell viability as verified by Annexin V/PI stainings (not shown). The *ex vivo* culture of human whole blood with BHT or BHA clearly reduced the sizes of the NETs formed by blood granulocytes in the presence of >90% plasma (**Figures 1C**,**D**). Ascorbic acid was even more potent and abolished NET formation under these conditions completely (**Figure 1E**).

### MSU INDUCE NET FORMATION IN HUMAN TISSUE SECTIONS

**FIGURE 2:**
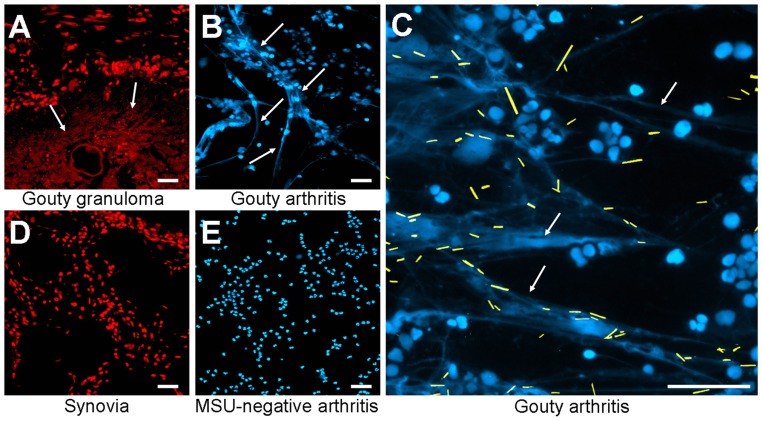
**Patients with MSU-induced inflammation display extranuclear DNA in tissue sections**. Revealed by DNA staining with propidium iodide, sections of gouty granulomas **(A)** display extranuclear DNA (arrows). Synovial fluids of patients suffering from gouty arthritis display extranuclear DNA (arrows) visualized by staining with DAPI **(B,C)**. All MSU crystals (artificially colored in yellow in **C**) were associated with NET structures **(C)**. Sections of human synovial control **(D)** and cytospins of synovial fluids from MSU-negative arthritis **(E)** exclusively showed nuclear DNA distribution. All experiments were performed at least five times. Scale bars, 100 μm.

The *in vitro* generation of NETs is well characterized, but the relevance for diseases requires *in vivo* data. Therefore, we analyzed whether MSU-associated NETs can also be found in human tissue sections. We analyzed gouty granulomas and surrounding non-granulomatous areas for the presence of extranuclear DNA structures, with the appearance of NETs generated in our *ex vivo* blood cultures. Extracellular DNA was detected by stainings with PI. As shown in **Figure 2A** extranuclear DNA is abundant in the granuloma (**Figure 2A**, lower part) whereas the chromatin is confined to the nuclei in the surrounding tissue (**Figure 2A**, upper part). Next we analyzed cytospins from synovial fluid from patients with gouty synovitis. Synovial sections and cytospins of MSU-negative arthritides served as controls. Again, extracellular thready chromatin was to be detected by DNA staining only in samples from patients suffering from gout (**Figures 2B**,**C**). In the controls the DNA was confined in the nuclei (**Figures 2D**,**E**). Due to the washing steps during the routine preparation of the tissue sections MSU crystals are usually dissolved and cannot be detected (**Figure 2A**). In contrast, the mild conditions used in the preparation of the cytospins from synovial fluids preserve the MSU. Interestingly, in the cytospins of the synovial fluids of patients with gouty arthritis the crystals are trapped within the extranuclear chromatin fibers (**Figure 2C**; MSU crystals artificially highlighted in yellow). Thus, in concordance with their role in the anti-bacterial responses, NETs reduce the uncontrolled spreading of pathogenic MSU crystals.

### MSU-INDUCED NETs DO NOT BIND TO OPSONINS

**FIGURE 3:**
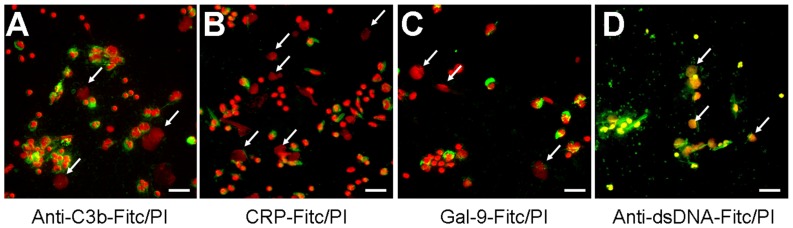
**The dead cells’ ligands C3b, CRP, and galectin-9 do not opsonize NETs**. **(A–D)** Isolated PMN were incubated with MSU for 5 h at 37°C and then fixed with 1% PFA. Cytospins were prepared and NETs visualized by PI staining (red). Anti-C3b **(A)**, CRP **(B)**, and galectin-9 **(C)** bind to PMN corpses but not to NETs (arrows). Antibodies against dsDNA as positive control **(D)** bind cell remnants and NETs (arrows). Scale bars, 50 μm.

The fast and efficient disposal of apoptotic and necrotic cell material is essential to avoid autoimmune reactions (Korns et al., 2011). NETs expose several nuclear autoantigens that are prototypical for the autoantigens targeted in systemic autoimmunity, e.g., SLE. NETs contain nucleic acids which are potent inducers of interferon-alpha, if they are recognized by endosomal TLR-3, -7, -8, and -9. Therefore, NETs may be considered hazardous waste that has to be disposed carefully. To clear toxic waste and pro-inflammatory cell remnants the phagocytes employ opsonizing immunoglobulins or complement proteins as well as other molecules bridging the phagocytes to their prey. If these molecules also opsonize/bind NETs structures was investigated by direct and indirect immune fluorescence analyses employing CRP-FITC/galectin-9-FITC/anti-dsDNA-FITC and anti-C3b-FITC, respectively. CRP is known to bind lysophosphatidylcholine on disturbed cellular membranes as well as nuclear components as histone H1 and small nuclear ribonucleoprotein (snRNP) particles (Jewell et al., 1993). Galectin-9 binds beta-galactosides exposed during the change of the glycomic profile during cell death and C3b is deposited during classical and alternative activation of the complement cascade. We therefore investigated the binding of CRP, galectin-9, and complement C3b to NET structures formed by isolated PMN cultured with 5 × 10^6^ cells/ml in response to MSU crystals (**Figure 3**). Surprisingly, C3b (**Figure 3A**), CRP (**Figure 3B**), and galectin-9 (**Figure 3C**) indeed bound to the cellular corpses of PMN but not to the NETs (see arrows). In contrast, antibodies against dsDNA bound both cell remnants and NETs (**Figure 3D**).

## DISCUSSION

MSU crystals are amongst the most potent NETosis inducing agents. Indeed, they represent the only stimuli that induce NETosis in whole blood *ex vivo* cultures or in neutrophils cultures the presence of pure plasma (this paper and Schorn et al., 2012). Here we showed that NET formation induced by MSU crystals is accompanied by ROS formation in neutrophils. This is in line with Fuchs et al. (2007) who reported that NETosis is dependent on the activation of NADPH oxidase and ROS production. Interestingly, diphenyleneiodonium, an inhibitor of the NADPH oxidase prevented NET formation. In our hands, the reduction of oxidative stress by the anti-oxidants BHT, BHA, and ascorbic acid abolished NETs formation as well. An ROS-dependent NET formation has also been shown for mast cells in response to PMA, H_2_O_2_, or bacterial pathogens (von Kockritz-Blickwede et al., 2008). Patients suffering from chronic granulomatous disease (CGD) carry a mutation in the genes coding for the NADPH oxidase and are, therefore, deficient in NETosis. The deficiency in the NET formation of patients with CGD may contribute to their immune deficient phenotype (Fuchs et al., 2007). Consequently, the restoration of NADPH function by gene therapy resulted in the reconstitution of NETs formation and neutrophil elimination of *Aspergillus nidulans* (Bianchi et al., 2009, 2011). As shown during sepsis intravascular NETs capture bacteria from the vasculature thus preventing dissemination. Consequently, blocking NETs formation results in increased spreading of bacteria to distant organs (McDonald et al., 2012). These findings underline the fundamental role of NETs in anti-microbial activity *in vivo*.

Analyses of synovial fluids and of tissue sections of patients suffering from gout revealed that NETs are also formed *in vivo*, especially during acute gouty flares and/or granuloma formation. Mitroulis et al. (2011) showed that the formation of NETs can be induced by the transfer of synovial fluid from inflamed tissues to control PMN. The occurrence of NETs has also been described in SLE, where it has been identified as a potential antigen for autoimmunity that trigger B cell activation (Lande et al., 2011). NETs have been shown to activate TLR-9 in dendritic cells and to prime T cells. Interestingly, autoantibodies found in SLE are often directed against both self-DNA and anti-microbial peptides located in NET structures (Nassberger et al., 1989; Fauzi et al., 2004). NETs are covered with HMGB1, which represents a highly active danger signal endowed with pro-inflammatory potential. Therefore, it has been speculated that after inflammatory responses the timely removal of NETs is essential to avoid exposure of intracellular self-antigens and, consequently, the challenge of immune tolerance (Hakkim et al., 2010). The DNA backbone of the NETs has been described to be performed by DNase-1 and patients with low serum DNase-1 activity have an increased risk to develop nephritis (Hakkim et al., 2010).

We found that the established dead cells’ opsonins C3b, galectin-9, and CRP decorated the residual dead cells’ corpses and opsonized these for disposal. Surprisingly, all three soluble pattern recognizing molecules spared the spread NET structures. However, the chromatin of the NETs is accessible to a monoclonal polyreactive DNA autoantibody. These results are in concordance with the observation of Fuchs et al. (2007) who showed that AxV only binds to phosphatidylserine restricted to the necrotic cell remnant but not to the dispersed chromatin containing NETs structures. Besides phosphocholine from disturbed mammalian membranes nuclear components as snRNP or histone H1 are common target structures for CRP binding (Janko et al., 2009). Surprisingly, CRP did not bind to the dispersed extracellular chromatin in the NETs. However, if the nuclear CRP targets are not included into the NETs, lost from the NETs structure due to low binding affinity or are modified during NETosis requires further investigations. In this context histone modifications prior to NETting might also play a role (Liu et al., 2012).

The binding of nuclear structures by opsonins as CRP has been discussed previously to efficiently mask self-antigens to preclude immune activation and to foster anti-inflammatory clearance (Marnell et al., 2005). Usually, the binding of CRP results in the recruitment of complement C1q and in complement activation. Previously it has been shown that complement C1q promotes the degradation of necrotic cell-derived chromatin (Gaipl et al., 2004). Remarkably, in a previous study C1q binding to NETs was detected, which inhibited the degradation of NETs by a still unknown mechanism, whereas anti-C1q antibodies reversed this effect (Leffler et al., 2012). In our hands, anti-dsDNA antibodies bound to the NETs and formed nucleic acid containing immune complexes similar to those generated in SLE by secondary necrotic cells and lupus typical autoantibodies (Munoz et al., 2010). In this context pro-inflammatory activities are exerted by antibodies that bind secondary necrotic cells either directly or via a bridging molecule like CRP (Janko et al., 2011). The fact that immune complexes can trigger NET formation (Kessenbrock et al., 2009) and NETs can form immune complexes may be involved in the vicious circle operating in patients with clearance deficiency or low DNase-1 activities (Munoz et al., 2009). If NET-IgG immune complexes harbor a similar pro-inflammatory potency like IgG-opsonized secondary necrotic cells (Meesmann et al., 2009) is currently investigated in our lab. Taking together, we consider NETs as a problematic prey especially in the presence of NET-binding autoantibodies.

## Conflict of Interest Statement

The authors declare that the research was conducted in the absence of any commercial or financial relationships that could be construed as a potential conflict of interest.

## Abbreviations

BETs: basophil extracellular traps
BHA: butylated hydroxyanisole
BHT: butylated hydroxytoluene
CRP: C-reactive protein
DAPI: 4′,6-diamidino-2-phenylindole
DCFH-DA: dichlorofluorescein-diacetate
EETs: eosinophil extracellular traps
FSc: forward scatter
Gal: galectin
IL: interleukin
MCETs: mast cell extracellular traps
MSU: monosodium urate
NADPH: nicotinamide adenine dinucleotide phosphate
NET: neutrophil extracellular trap
PAD4: protein arginine deiminase 4
PFA: paraformaldehyde
PI: propidium iodide
PMN: polymorphonuclear neutrophils
ROS: reactive oxygen species
snRNP: small nuclear ribonucleoprotein
SSc: side scatter
